# Availability of national policies, programmes, and survey‐based coverage data to track nutrition interventions in South Asia

**DOI:** 10.1111/mcn.13555

**Published:** 2023-08-17

**Authors:** Sumanta Neupane, Manita Jangid, Samuel P. Scott, Sunny S. Kim, Zivai Murira, Rebecca Heidkamp, Bianca Carducci, Purnima Menon

**Affiliations:** ^1^ Poverty, Health, and Nutrition Division International Food Policy Research Institute New Delhi Delhi India; ^2^ World Food Program Rome Italy; ^3^ International Food Policy Research Institute Washington District of Columbia USA; ^4^ UNICEF Regional Office for South Asia Kathmandu Nepal; ^5^ Johns Hopkins Bloomberg School of Public Health Baltimore Mayland USA

**Keywords:** intervention coverage, nutrition interventions, nutrition policy, nutrition programmes, South Asia

## Abstract

Progress to improve nutrition among women, infants and children in South Asia has fallen behind the pace needed to meet established global targets. Renewed political commitment and monitoring of nutrition interventions are required to improve coverage and quality of care. Our study aimed to assess the availability of national nutrition policies, programmes, and coverage data of nutrition interventions for women, children, and adolescents in eight countries in South Asia. We reviewed relevant policy and programme documents, examined questionnaires used in the most recent rounds of 20 nationally representative surveys, and generated an evidence gap map on the availability of policies, programmes, and survey data to track progress on coverage of globally recommended nutrition interventions. Current policies and programmes in South Asian countries addressed almost all the recommended nutrition interventions targeted at women, children, and adolescents. There was a strong policy focus in all countries, except Maldives, on health system platforms such as antenatal and postnatal care and child growth and development. Survey data on nutrition intervention coverage was most available in India and Nepal, while Bangladesh and Bhutan had the least. Though countries in South Asia have committed to national nutrition policies and strategies, national surveys had substantial data gaps, precluding progress tracking of nutrition intervention coverage. Greater attention and effort are needed for multisectoral collaboration to promote and strengthen nutrition data systems.

## Key messages

- Most South Asian countries had several policies and programmes in place which considered or addressed nutrition interventions across the life course.
- Nationally representative surveys provide coverage data for evidence‐based recommendations to improve policy and programme actions.
- Significant coverage data gaps remain in nationally representative surveys around counselling during pregnancy, interventions targeted for newborns, infant, and young child feeding, and counselling on and identification and treatment of severe and moderate acute malnutrition.
- Mobilising political commitment and increasing multisectoral collaboration are required for an enabling environment in support of nutrition data systems in South Asia.

## Graphical abstract

Growing rates of malnutrition, both under‐ and overnutrition, continue to threaten the South Asia region. Our study examined national policies, programmes, and data availability on nutrition interventions in eight South Asian countries. Results revealed a strong policy focus, but data gaps limit progress tracking. Multisectoral collaboration and strengthened nutrition data systems are required to overcome nutrition problems in the region.

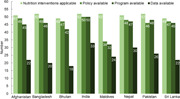

## 1 INTRODUCTION

Over recent decades, countries in South Asia have undergone profound demographic and epidemiologic transitions, drastically changing the patterns of disease burden (Vicziany, 2021). Growing rates of malnutrition, both under‐ and overnutrition, continue to threaten the region's economic and social growth. According to United Nations Children's Fund (UNICEF) estimates, at least 30% of children under‐5 years are stunted and 14% are wasted in South Asia, bearing the highest prevalence globally (UNICEF, WHO, The World Bank, 2021). Equally alarming, the prevalence of micronutrient deficiencies (Stevens, Beal, et al., 2022), anaemia in women of reproductive age (Owais et al., 2021; Stevens, Paciorek, et al., 2022), and noncommunicable diseases among adults are increasingly contributing to morbidity and mortality in the region (World Health Organization, 2022b). As a result, reducing malnutrition has become an international development priority in South Asia, with political momentum and alignment of country strategies to meet the World Health Assembly 2025 targets and sustainable development goals (SDGs) by 2030 (Sachs et al., 2021).

At a regional level, the South Asian Association for Regional Cooperation (SAARC), provides a platform for countries to work collaboratively, advocate, and work in partnership to address common issues such as nutrition that transcend political and geographical borders (South Asian Association For Regional Cooperation, 2014). In 2014, SAARC developed the Regional Action Framework for Nutrition, which highlights that optimal nutritional outcomes for children in the region can be achieved by building an enabling environment and supporting the scale‐up of sustainable nutrition interventions (South Asian Association For Regional Cooperation, 2014). Four strategic pillars were proposed including: (i) soliciting political commitment to improve nutrition governance, strengthen programme planning, and implement multisectoral policies and plans; (ii) scaling up cost‐effective evidence‐based, sustainable nutrition‐specific and nutrition‐sensitive interventions for all; (iii) increasing human and institutional capacity to manage nutrition programmes nationally and subnationally; and (iv) increasing effectiveness and accountability of nutrition intervention implementation stakeholders through monitoring and knowledge translation mechanisms (South Asian Association For Regional Cooperation, 2014). Within member countries, policies and programmes need to be in place to support these nutritional actions (Pike et al., 2021; World Health Organization, 2019).

Effective nutrition interventions, including a combination of direct (nutrition‐specific) and indirect (nutrition‐sensitive) interventions that are delivered within and outside the health care sector, are required to address malnutrition (Keats et al., 2021; Vaivada et al., 2022). These strategies must work across the continuum of care to reduce disparities and reach at‐risk and neglected populations such as women, children, and adolescents (Keats et al., 2021; Vaivada et al., 2022). Key interventions across the life course include counselling and support for exclusive breastfeeding and appropriate complementary feeding, antenatal care, school food policies and programmes, and regulations and enforcement around marketing for unhealthy foods and breast milk substitutes (Keats et al., 2021; Vaivada et al., 2022). Research on which platforms are best suited to deliver packages of care to beneficiaries that are both age‐appropriate and timely is ongoing (Janmohamed et al., 2019). Importantly, there is much dialogue on improving integration and scale‐up of nutrition interventions within health systems (Holschneider et al., 2022; Salam et al., 2019; Subandoro et al., 2021). These various policy and programmatic decisions related to the design, adaptation, and implementation of comprehensive evidence‐based nutrition actions then must be informed continually by information about intervention coverage and performance at the population level.

However, studies from low‐ and middle‐income countries have shown that nutrition interventions are often either not delivered consistently or are not of sufficient quality during critical maternal and child health contact points (Gillespie et al., 2019; Menon et al., 2014). This shortcoming is in part due to poor data integration, reliability, availability, and granularity in standardised surveys that collect nutrition data such as Demographic and Health Surveys (DHS), National Nutrition Surveys (NNS) or Multiple Indicator Cluster Survey (MICS). This, in turn, has hindered the analysis and interpretation of actionable coverage indicators to improve the delivery and quality of interventions (Amouzou et al., 2019; Gillespie et al., 2019).

The objective of this paper was to assess the availability of and gaps between policies, programmes, and survey‐based coverage data on essential nutrition interventions in eight South Asian countries (Afghanistan, Bangladesh, Bhutan, India, Maldives, Nepal, Pakistan, Sri Lanka).

## 2 METHODS

This study was conducted under the Data for Decisions to Expand Nutrition Transformation (DataDENT) initiative, which aims to transform the availability and use of nutrition data by addressing gaps in nutrition measurement and advocating for stronger nutrition data systems (Institute For International Programmes, International Food Research Policy Institute, Results For Development Institute, 2022). The study was conducted in close collaboration with the UNICEF Regional Office of South Asia (ROSA) and its country offices in the region, particularly to gather all relevant documents and validate findings along the document and data review process.

### 2.1 Identification of globally recommended nutrition interventions

We generated a list of nutrition interventions based on global recommendations from (1) World Health Organization (WHO), (2) Every Woman Every Child (EWEC), and (3) Lancet series on maternal and child nutrition 2021 (Every Woman, Every Child, 2016; Keats et al., 2021; World Health Organization, 2004, 2014, 2015, 2016, 2018, 2019). We included interventions recommended during five stages throughout the life course—adolescence, preconception, pregnancy, postpartum/lactation, and early childhood. These included mostly direct and few indirect nutrition interventions provided to beneficiaries and delivered through health and other sector platforms. For instance, antenatal care is a health sector platform for providing micronutrient supplementation and nutrition counselling to pregnant women. Likewise, parturition at a health facility and attendance by a skilled health worker are necessary to deliver interventions such as delayed cord clamping, assessment of birthweight, support for early initiation of breastfeeding, and immediate skin‐to‐skin contact. However, interventions targeted at adolescents are often delivered through schools (education sector platform). In total, we identified 54 globally recommended nutrition interventions (46 by WHO, 6 by EWEC, and 22 Lancet Nutrition Series 2021), four during adolescence, four during preconception, 18 during pregnancy, 12 during delivery and lactation, and 16 during early childhood (Table 1).

**Table 1:** List of 54 recommended nutrition interventions by life stage.

Adolescence (*n* = 4)	Preconception (*n* = 4)	Pregnancy (*n* = 18)	Delivery and lactation (*n* = 12)	Early childhood (*n* = 16)
1. Daily or intermittent iron and folic acid (IFA)^a^ supplementation.^b^ 2. Preventive deworming.^a^^,^ c 3. Food supplementation. 4. Counselling/education on healthy diets.	5. Daily or intermittent IFA supplementation.^a^^,^ ^b^ 6. Preventive deworming.^a^^,^ c 7. Contraception. 8. Salt fortification (iodine supplementation).	9. Antenatal care (ANC) screening by a trained provider. 10. ANC screening in first trimester by a trained provider. 11. At least four ANC visits. 12. Energy and protein dietary supplementation.^a^^,^ d 13. Daily or intermittent IFA supplementation.^a^ 14. Vitamin A supplementation.^a^^,^ e 15. Calcium supplementation.^a^ 16. Iron‐containing micronutrient powder (MNP) supplementation.^a^^,^ f 17. Preventive deworming.^a^^,^ g 18. Tetanus toxoid vaccination. 19. Nutritional counselling on healthy diet.^a^^,^ d 20. Weight monitoring. 21. Advice about weight after weighing. 22. Advice on consuming calcium. 23. Advice on consuming IFA. 24. Advice on consuming additional food. 25. Advice on birth preparedness. 26. Advice on exclusive breastfeeding.	27. Institutional birth. 28. Skilled birth attendant. 29. Optimal timing of (delayed) cord clamping.^a^ 30. Assessment of birthweight 31. Support for exclusive breastfeeding and skin‐to‐skin contact.^a^ 32. Optimal feeding of low‐birthweight (LBW) infants. 33. Counsel mothers of LBW infants on kangaroo mother care.^a^ 34. Postnatal care for babies. 35. Postnatal care for women. 36. Breastfeeding counselling.^a^ 37. Daily or intermittent IFA supplementation.^a^^,^ h 38. Food supplementation for malnourished lactating women.	39. Breastfeeding counselling.^a^ 40. Counselling on appropriate complementary feeding.^a^ 41. Food supplementation for complementary feeding.^a^ 42. Iron‐containing MNP.^a^^,^ i 43. Daily or intermittent IFA supplementation.^a^^,^ j 44. Zinc during diarrhoea.^a^ 45. Oral rehydration salt during diarrhoea.^a^ 46. Vitamin A supplementation.^a^^,^ k 47. Preventive deworming.^a^^,^ l 48. Growth monitoring.^a^ 49. Counselling on nutritional status.^a^ 50. Identification of severely or moderately underweight.^a^ 51. Inpatient management of severe acute malnutrition (SAM).^a^ 52. Outpatient management of SAM.^a^ 53. Management of moderate acute malnutrition.^a^ 54. Immunisation.

^a^ Recommended WHO under essential nutrition actions mainstreaming nutrition through the life course.

^b^ Intermittent if anaemia prevalence is more than 20% and daily if anaemia prevalence is greater than 40% among nonpregnant women.

^c^ If prevalence of any soil‐transmitted helminth infection is 20% or higher among adolescents 11–19 years.

^d^ If underweight prevalence among women is more than 20%.

^e^ Where 5% or more of women have a history of night blindness during pregnancy in the past 3–5 years, or if 20% or more of pregnant women have vitamin A deficiency.

^f^ In settings with a high prevalence of nutritional deficiencies.

^g^ Where pregnant women have a 20% or higher prevalence of infection with hookworm or *Trichuris trichiura* infection AND a 40% or higher prevalence of anaemia.

^h^ With a 20% or higher population prevalence of gestational anaemia.

^i^ Where the prevalence of anaemia in children under 5 years of age is 20% or more.

^j^ Daily if anaemia prevalence among children aged 6–59 months is 40% or more; intermittent for children aged 24–59 months if anaemia prevalence among this group is 20% or more.

^k^ Where the prevalence of night blindness is 1% or more in children aged 24–59 months, or the prevalence of vitamin A deficiency is 20% or higher in infants and children aged 6–59 months.

^l^ Living in areas where the baseline prevalence of any soil‐transmitted infection is 20% or higher among children aged 12 months and older.

### 2.2 Data collection

To identify the existing policies and programme documents related to nutrition from eight countries in South Asia (Afghanistan, Bangladesh, Bhutan, India, Maldives, Nepal, Pakistan, and Sri Lanka), we searched online and contacted the UNICEF ROSA and its country offices. We maintained a broad definition of policies and programmes to ensure that all relevant documents were captured in the search. Documents included any direct or indirect nutrition policy, strategy, legislation, regulation, or guidelines applicable at the national, state, or regional levels that were endorsed by the government. If a recommended nutrition intervention was addressed by a policy, directly or indirectly, we assessed if a programme was in place, which means operational as of February 2022. We identified policy and programme gaps (or the lack of a policy or a programme) for interventions that are applicable based on the epidemiological context of each country. For example, iron and folic acid (IFA) supplementation for adolescents is recommended by WHO only if the anaemia prevalence among women of reproductive age is more than 20%; therefore, we did not consider IFA supplementation for adolescents as a recommended intervention in a country where anaemia among women was <20%. We determined the applicability of interventions in each country based on the prevalence estimates from nationally representative population‐based surveys and Global Nutrition Report country profiles, recommendation for all settings, and/or existence of the programme in the country (Supporting Information: Table S1).

Then, to examine the availability of intervention coverage data, we reviewed questionnaires used in the most recent rounds of 20 nationally representative population‐based surveys (i.e., DHS, NNS, MICS, and micronutrient national surveys conducted in the eight countries between 2012 and 2019). We did not include routine administrative data or programme monitoring data as part of this study because a separate review of examining these data sources in South Asia was conducted by UNICEF. The review of nutrition policies, programmes, and survey data were conducted between January 2021 and February 2022.

### 2.3 Data extraction and synthesis

To generate an evidence gap map on the existence of policies and programmes, information from policy and programme documents related to the applicable interventions was extracted for each of the eight countries. We extracted the name of the policy document, the name of programme(s) which addressed one or more nutrition interventions, and any programme implementation guidelines. The final data results were synthesised to generate an evidence gap map of the policies, programmes, and survey‐based coverage data.

## 3 RESULTS

### 3.1 Policy, programme, and data gaps for interventions across the life course

A total of 104 policy and programme documents were reviewed and included in this study—Afghanistan (10 documents), Bangladesh (16), Bhutan (9), India (17), Maldives (7), Nepal (13), Pakistan (10), and Sri Lanka (22) (Supporting Information: Table S2). We also reviewed questionnaires used in the most recent rounds of 20 nationally representative population‐based surveys—Afghanistan (two surveys), Bangladesh (3), Bhutan (2), India (2), Maldives (2), Nepal (2), Pakistan (2), and Sri Lanka (5) (Supporting Information: Table S3).

**Figure 1:**
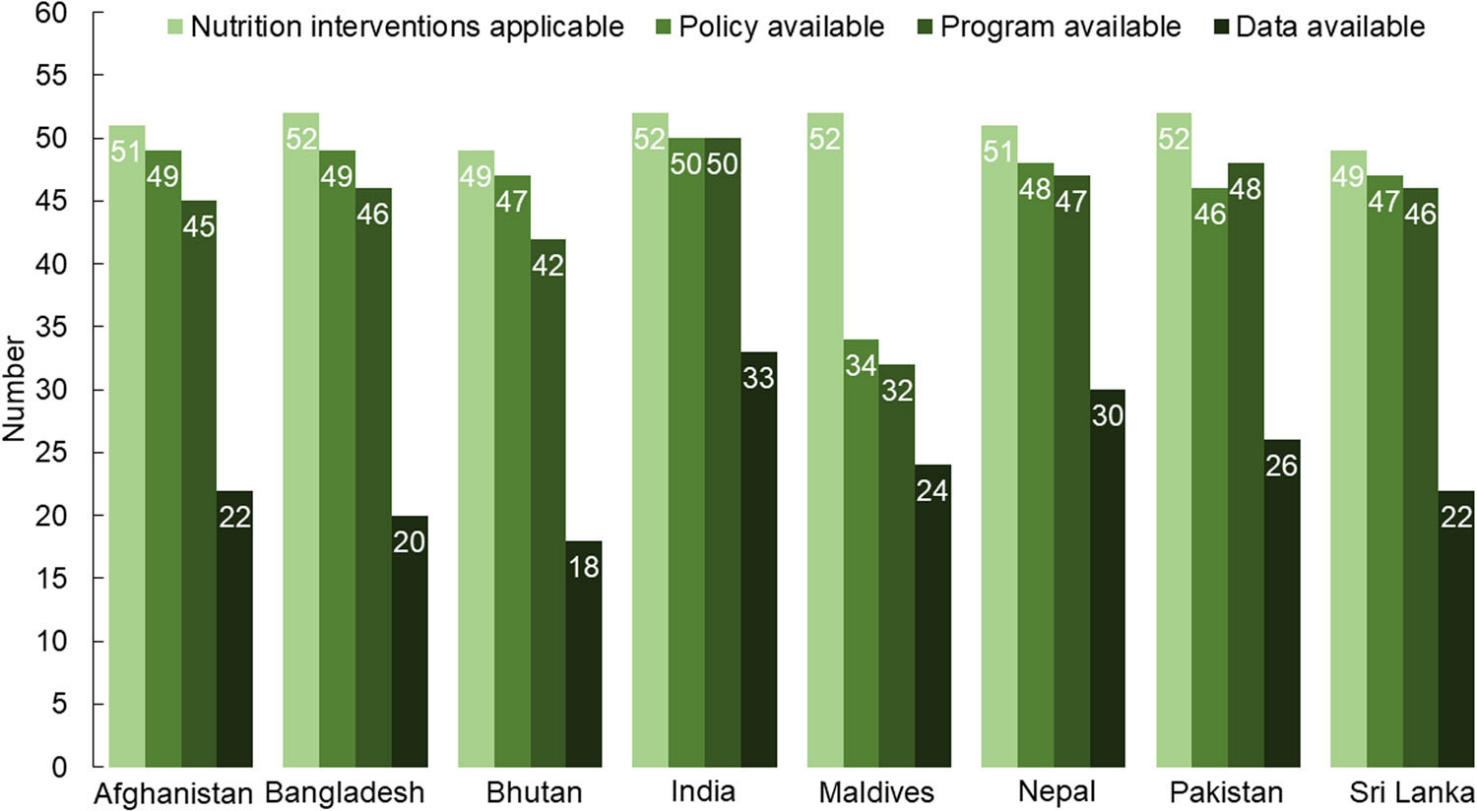
Numbers of nutrition interventions applicable and policy, programme, and data available by country.

Of the total 54 recommended interventions, the number of applicable interventions ranged from 52 in Bangladesh, India, and Pakistan to 49 in Bhutan (Figure 1). Fifty‐two nutrition interventions were applicable in the Maldives, but policies in the country only covered 34 of these interventions (policy gap of 16), programmes covered 32 (programme gap of 18), and data were available for 24 (data gap of 26) (Figure 1). Bangladesh and Bhutan had the largest data gaps of 32 and 31, respectively. Although India presented the smallest policy/programme and data gaps, there were still 19 interventions for which coverage data were not available in either of the two national surveys.

The most prominent policy and programme gaps were for food supplementation during adolescence; IFA supplementation and deworming during preconception; calcium supplementation and advice on consuming calcium during pregnancy; food supplementation for complementary feeding, IFA supplementation, and iron‐containing micronutrient powder (MNP) during early childhood. Data gaps existed mostly for IFA supplementation and deworming during adolescence and preconception; food supplementation, calcium supplementation, and advice on consuming IFA and calcium during pregnancy; interventions targeted for newborns, IFA supplementation, and food supplementation during delivery and postpartum; counselling on infant and young child feeding (IYCF), including both breastfeeding and complementary feeding practices, food supplementation for complementary feeding, IFA supplementation, iron‐containing MNP, and treatment and management of severe acute malnutrition (SAM) and moderate acute malnutrition (MAM) during early childhood.

#### 3.1.1 During adolescence

Bangladesh, Bhutan, India, Nepal, and Pakistan had no policy and programme gap during adolescence (Table 2). One out of four interventions were not available in Afghanistan, Maldives, and Sri Lanka, and two out of four in Maldives. Data gap during adolescence was 100% in Afghanistan, Bangladesh, Bhutan, Maldives, and Pakistan. Surveys contained coverage data on three interventions in Nepal, two in India, and one in Sri Lanka.

**Table 2:** Availability of policy, programme, and data on nutrition interventions recommended from adolescence through pregnancy by country.

Life stages	Nutrition interventions	AF			BD			BH			IN			ML			NP			PK			SL		
		Po	Pr	D	Po	Pr	D	Po	Pr	D	Po	Pr	D	Po	Pr	D	Po	Pr	D	Po	Pr	D	Po	Pr	D
Adolescence	1. Daily or intermittent IFA supplementation																								
	2. Preventive deworming																								
	3. Food supplementation																								
	4. Counselling/education on healthy diets																								
	*% Of applicable interventions available*	*75*	*75*	*0*	*100*	*100*	*0*	*100*	*100*	*0*	*100*	*100*	*50*	*50*	*25*	*0*	*100*	*100*	*75*	*100*	*100*	*0*	*66*	*66*	*33*
Preconception	5. Daily or intermittent IFA supplementation																								
	6. Preventive deworming																								
	7. Contraception																								
	8. Salt fortification (iodine supplementation)																								
	*% Of applicable interventions available*	*100*	*50*	*50*	*75*	*75*	*50*	*100*	*33*	*66*	*100*	*100*	*50*	*66*	*33*	*66*	*100*	*66*	*66*	*50*	*50*	*50*	*100*	*66*	*66*
Pregnancy	9. Any ANC screening by a trained provider																								
	10. ANC screening by a trained provider during first trimester																								
	11. Four or more ANC visits																								
	12. Energy and protein dietary supplementation																								
	13. Daily or intermittent IFA supplementation																								
	14. Vitamin A supplementation																								
	15. Calcium supplementation																								
	16. Iron‐containing MNP supplementation																								
	17. Preventive deworming																								
	18. Tetanus toxoid vaccination																								
	19. Nutritional counselling on healthy diet																								
	20. Weight monitoring																								
	21. Advice about weight after weighing																								
	22. Advice on consuming calcium																								
	23. Advice on consuming IFA																								
	24. Advice on consuming additional food																								
	25. Advice on birth preparedness																								
	26. Advice on exclusive breastfeeding																								
	*% Of applicable interventions available*	*100*	*93*	*60*	*100*	*100*	*37*	*100*	*94*	*44*	*100*	*100*	*75*	*80*	*80*	*73*	*88*	*88*	*50*	*100*	*100*	*68*	*100*	*100*	*56*

*Note*: 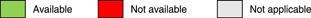

Abbreviations: AF, Afghanistan; ANC, antenatal care; BG, Bangladesh; BH, Bhutan; D, data; IFA, iron and folic acid; IN, India; ML, Maldives; MNP, micronutrient powder; NP, Nepal; PK, Pakistan; Po, policy; Pr, programme; SL, Sri Lanka.

#### 3.1.2 During preconception

We did not find any policy gap during preconception in Afghanistan, Bhutan, India, Nepal, and Sri Lanka (Table 2). There was no programme gap in India; however, in Bhutan and Maldives, only one out of three interventions was addressed by programmes. In all countries, at least 50% of the interventions had survey‐based coverage data.

#### 3.1.3 During pregnancy

A total of 18 interventions were recommended during pregnancy; 16 were applicable in five countries and 15 in three countries (Table 2). Policies and programmes in Bangladesh, India, Pakistan, and Sri Lanka addressed all recommended interventions. In Afghanistan and Bhutan, there was no policy gap, and programmes did not address one intervention. In Maldives and Nepal, policy and programme gaps were three and one interventions, respectively. We observed a large data gap during pregnancy; only 37% of the interventions in Bangladesh and 44% in Bhutan had coverage data in the surveys. Surveys in India had data on 75% of the interventions, which was the highest among all eight countries.

#### 3.1.4 During delivery and lactation

All 12 recommended interventions during delivery and lactation were applicable in the eight countries (Table 3). Six countries had no policy and programme gap. In Bhutan and Maldives, 92% and 75% of the interventions, respectively were addressed by policies and programmes. Percentage of interventions that had data ranged between 42% in Afghanistan and 58% in India and Nepal.

**Table 3:** Availability of policy, programme, and data on nutrition interventions recommended from delivery through childhood by country.

Life stages	Nutrition interventions	AF			BD			BH			IN			ML			NP			PK			SL		
		Po	Pr	D	Po	Pr	D	Po	Pr	D	Po	Pr	D	Po	Pr	D	Po	Pr	D	Po	Pr	D	Po	Pr	D
Delivery and lactation	1. Institutional birth																								
	2. Skilled birth attendant																								
	3. Optimal timing (delayed) of umbilical cord clamping																								
	4. Assessment of birthweight																								
	5. Support for EBF and immediate skin‐to‐skin contact																								
	6. Optimal feeding of LBW infants																								
	7. Counselling of mothers of LBW infants on KMC																								
	8. PNC care for babies (3 days, 7 days, and 6 weeks after delivery)																								
	9. PNC for women (3 days, 7 days, and 6 weeks after delivery)																								
	10. Breastfeeding counselling																								
	11. IFA supplementation																								
	12. Food supplementation for malnourished lactating women																								
	*% Of applicable interventions available*	*100*	*100*	*42*	*100*	*100*	*50*	*92*	*92*	*50*	*100*	*100*	*58*	*75*	*75*	*50*	*100*	*100*	*58*	*100*	*100*	*50*	*100*	*100*	*50*
Childhood	13. Breastfeeding counselling																								
	14. Counselling on appropriate complementary feeding																								
	15. Food supplementation for complementary feeding																								
	16. Iron‐containing MNP supplementation																								
	17. Daily IFA supplementation																								
	18. Zinc supplementation during diarrhoea																								
	19. ORS during diarrhoea																								
	20. Vitamin A supplementation																								
	21. Preventive deworming																								
	22. Growth monitoring (weight assessment)																								
	23. Counselling on nutritional status																								
	24. Identification of severe or moderate underweight																								
	25. Inpatient management of SAM																								
	26. Outpatient management of SAM																								
	27. Management of MAM																								
	28. Immunisation																								
	*% Of applicable interventions available*	*94*	*87*	*38*	*69*	*62*	*37*	*93*	*80*	*27*	*87*	*87*	*62*	*56*	*56*	*25*	*94*	*94*	*56*	*87*	*87*	*44*	*93*	*93*	*33*

*Note*: 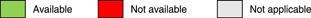

Abbreviations: AF, Afghanistan; BG, Bangladesh; BH, Bhutan; D, data; EBF, exclusive breastfeeding; IBF, iron and folic acid; IN, India; KMC, kangaroo mother care; LBW, low‐birthweight; MAM, moderate acute malnutrition; ML, Maldives; MNP, micronutrient powder; NP, Nepal; ORS, oral rehydration salt; PK, Pakistan; PNC, postnatal care; Po, policy; Pr, programme; SAM, severe acute malnutrition; SL, Sri Lanka.

#### 3.1.5 During early childhood

Out of the total 16 recommended interventions during early childhood, policies and programmes in six countries addressed more than 80% of them (Table 3). Policies and programmes in Maldives addressed 56% of the interventions, which were the lowest among all eight countries. The data gap was highest in Bhutan and Maldives; only 27% of the interventions in Bhutan and 25% in Maldives had survey data. Data were available for less than 50% of the interventions in Afghanistan, Bangladesh, Pakistan, and Sri Lanka. India and Nepal had data on 62% and 56% of the interventions, respectively.

### 3.2 Country‐specific policy, programme, and data gaps

#### 3.2.1 Afghanistan

Afghanistan's policies and programmes did not address food supplementation during adolescence and during early childhood (Tables 2 and 3). Programmes did not address IFA supplementation and deworming during preconception, calcium supplementation during pregnancy, and IFA supplementation during early childhood. Out of the 45 nutrition interventions that Afghanistan's policies and programmes addressed, surveys provided coverage data on 22 interventions; this included two aimed at women during preconception, nine during pregnancy, five during delivery and postpartum, and six interventions for early childhood (Figure 1).

None of the surveys contained data on interventions during adolescence. For interventions during pregnancy, data were missing on advice about gestational weight gain after weighing; advice on consuming calcium, IFA, and additional food; and advice on birth preparedness. For interventions during delivery and at postpartum, data were not available for early initiation of breastfeeding, care of low‐birthweight babies, breastfeeding counselling, IFA supplementation, or food supplementation for malnourished lactating women. There was no coverage data on counselling on nutritional status or MAM management during early childhood.

#### 3.2.2 Bangladesh

Bangladesh's policies and programmes did not address deworming during preconception or IFA supplementation during early childhood. Food supplementation for complementary feeding was addressed by policies, but not by programmes (Tables 2 and 3). Of the 49 nutrition interventions that Bangladesh's policies and programmes addressed, surveys provided coverage data on 20 interventions; this included two aimed at women during preconception, six during pregnancy, six during delivery and at postpartum, and six interventions for early childhood (Figure 1).

None of the surveys contained data on nutrition interventions for adolescents. For interventions during preconception, data were missing on IFA supplementation. For interventions during pregnancy, data were missing on maternal nutrition counselling, calcium supplementation, preventive deworming, advice on consuming calcium, advice on consuming IFA, advice on birth preparedness, and advice on exclusive breastfeeding. For women during delivery and in the postnatal period, survey data were not available for support on breastfeeding and skin‐to‐skin contact, optimal feeding of low‐birthweight babies, counselling of mothers of low‐birthweight infants on kangaroo mother care (KMC), breastfeeding counselling, and food supplementation for malnourished lactating women.

#### 3.2.3 Bhutan

Bhutan's policies and programmes did not address food supplementation for malnourished lactating women during delivery and IFA supplementation during early childhood. Policies addressed five interventions for which there were no programmes to implement it: IFA supplementation and preventive deworming during preconception, advice about gestational weight gain after weighing during pregnancy, outpatient SAM and MAM management during early childhood. Of the 42 nutrition interventions that Bhutan's policies and programmes addressed, survey data were available on coverage of 18 interventions; this included one intervention during preconception, six during pregnancy, six during delivery and at postpartum, and four for early childhood (Figure 1).

None of the surveys contained data on interventions for adolescence (Table 3). Regarding interventions during pregnancy, data for six out of 14 interventions was available. Data were not available for IFA supplementation, deworming, and food supplementation during adolescence; calcium supplementation, deworming, weight monitoring, and nutrition counselling during pregnancy; newborn care during delivery and the postnatal period; and IYCF counselling, growth monitoring, immunisation, and identification and management of SAM and MAM during early childhood. Data for six of 11 interventions during delivery and at postpartum was available. Data were not available on timing of cord clamping, support for early initiation of breastfeeding, optimal feeding of low‐birthweight infants, counselling of mothers on KMC, and IFA supplementation. For early childhood, data for four of the 12 interventions was available. None of the surveys contained data on counselling on breastfeeding/complementary feeding, iron‐containing MNP, and inpatient management of SAM.

#### 3.2.4 India

In India, policies and programmes did not address iron‐containing MNP supplementation and outpatient management of SAM during early childhood. Of the 50 nutrition interventions addressed by policies and programmes, 33 interventions had coverage data in the surveys. During adolescence, IFA supplementation had no data, and during preconception, there was no data on IFA supplementation and preventive deworming. During delivery and early childhood, five and seven interventions respectively had no data.

#### 3.2.5 Maldives

Many nutrition interventions were not addressed by either policies or programmes in the Maldives. These interventions include: IFA supplementation, deworming, and food supplementation during adolescence; IFA supplementation and deworming during preconception; calcium supplementation, advice on consuming calcium supplementation and on preventive deworming during pregnancy; optimal timing (delayed) of umbilical cord clamping; IFA and food supplementation for malnourished lactating women and food supplementation for complementary feeding; and, during early childhood, iron‐containing MNP, IFA supplementation, identification of severe or moderate underweight, inpatient management of SAM, outpatient management of SAM, and management of MAM. Of the 32 nutrition interventions addressed in policies and programmes, there was coverage data on 24 interventions; this included one action targeting women during preconception, 10 interventions during pregnancy, six interventions during delivery and in the postnatal period, and five interventions during early childhood (Figure 1).

Surveys did not contain data on advising on the consumption of IFA during pregnancy; support for early breastfeeding and immediate skin‐to‐skin contact, advising on optimal feeding of low‐birthweight infants and counselling on KMC during delivery and the postnatal period; IYCF counselling, growth monitoring, and counselling after growth monitoring during early childhood (Table 3).

#### 3.2.6 Nepal

No policies or programmes in Nepal addressed calcium supplementation and advice on consuming calcium during pregnancy, or IFA supplementation during early childhood. Programmes did not address IFA supplementation during preconception. Of the 46 nutrition interventions addressed in policies and programmes, surveys provided coverage data on 30 interventions. These included two each during adolescence and preconception, eight for pregnant women, seven during delivery and the postnatal period, and eight for early childhood (Figure 1).

Surveys did not include data on food supplementation during adolescence, energy and protein dietary supplementation, weight monitoring, or on the various types of counselling (Table 3). There was no data on nutrition interventions during delivery and in the postnatal period, delayed umbilical cord clamping, support for breastfeeding and immediate skin‐to‐skin contact, or interventions for low‐birthweight newborns. There were also no data on interventions during early childhood, including IYCF counselling, counselling after growth monitoring, or inpatient management of SAM.

#### 3.2.7 Pakistan

Policies and programmes in Pakistan did not address IFA supplementation and deworming during preconception, and food supplementation and IFA supplementation during early childhood. Of the 48 nutrition interventions addressed in policies and programmes, surveys provided coverage data on 26 interventions: two during preconception, 11 during pregnancy, six during delivery and the postnatal period, and six during early childhood (Figure 1).

None of the surveys contained data on interventions during adolescence, data on advice about gestational weight gain after weighing, advice on consuming calcium and IFA, or advice on birth preparedness (Table 3). There was no data on interventions during delivery and the postnatal period, including delayed umbilical cord clamping, support for early breastfeeding and immediate skin‐to‐skin contact, interventions for low‐birthweight infants, IFA supplementation, and food supplementation. Surveys did not contain data on counselling IYCF, growth monitoring, and counselling, and identification and management of SAM and MAM.

#### 3.2.8 Sri Lanka

Sri Lanka's policies and programmes did not address food supplementation during adolescence or for complementary feeding during early childhood. Programmes did not address IFA supplementation during preconception. Of the 46 nutrition interventions that were addressed by policies and programmes, surveys provided coverage data on 23 interventions (Figure 1).

There was no data on nutrition counselling for pregnant women, including advice on a healthy diet; counselling about gestational weight gain after weighing; advice on consuming calcium, IFA, and additional food; and counselling on birth preparedness and exclusive breasting (Table 3). Surveys did not contain data on various interventions during delivery and in the postnatal period, including delayed umbilical cord clamping, support for early breastfeeding and immediate skin‐to‐skin contact, optimal feeding of low‐birthweight infants, counselling on KMC, breastfeeding counselling, and IFA supplementation. For interventions during childhood, data were not available on IYCF counselling, MNP supplementation, growth monitoring and counselling, and identification and management of SAM and MAM.

## 4 DISCUSSION

The heightened visibility of malnutrition in all its forms, particularly within the millennium development goals and SDGs, has accelerated the tracking of global progress, identification of evidence‐based interventions, and advocacy for national policy reforms to incorporate nutrition as a key priority in agenda‐setting (International Food Research Policy Institute, 2015). Despite this, half of the countries in South Asia are off track to meet all 10 global nutrition targets (Afghanistan, Bhutan, India, Maldives), while two countries are on track for one target (Sri Lanka and Nepal) or two targets (Bangladesh and Pakistan) (Development Initiatives, 2021). Our review illustrated that apart from the Maldives, all other South Asian countries had a significant number of policies and programmes in place which considered or addressed multiple interventions across the life course. This included a strong policy focus on nutrition intervention delivery through health system service delivery platforms such as well‐care contacts during and after pregnancy (i.e., antenatal care visits, skilled birth attendants, IFA supplementation, deworming, breastfeeding, and IYCF counselling), and child growth and development (i.e., management of diarrhoea, growth monitoring, immunisation, and identification and management of SAM and MAM). With regard to data gaps for the recommended nutrition interventions, we observed that India and Nepal had the most available coverage data, reported earlier (Nguyen et al., 2022), while Bhutan had the least. Data gaps were most common in counselling during pregnancy, interventions targeted at newborns, IYCF counselling, and identification and treatment of SAM and MAM.

Policies and legislation, together with corresponding strategies, plans, budgets, coordination structures, and monitoring mechanisms, help create the conditions for an enabling environment for nutrition. National and subnational governments should take stock of missing elements in the enabling environment to better understand and address barriers and bottlenecks to implementing programmes at scale across all systems (Torlesse et al., 2022).

Some nutrition intervention policies and programmes may necessitate progress tracking through other data sources, aside from population‐based surveys. For example, governments have been called upon repeatedly to adopt and implement the World Health Organization International Code of Marketing of Breast Milk Substitutes (World Health Organization, 2022a) and subsequent Resolutions by the World Health Assembly to protect and promote breastfeeding, (i.e., World Health Organization Set of Recommendations on the Marketing of Foods and Nonalcoholic Beverages to Children (World Health Organization, 2010) and the United Nations Convention on the Rights of the Child) to protect mothers and children from exposure to unhealthy food and beverage marketing (Ngqangashe et al., 2022). However, introducing nutrition legislation that restricts marketing of unhealthy foods and beverages is both politically and technically difficult in terms of routinely monitoring and enforcing compliance. In reviewing national strategies in South Asia, all countries, except Bhutan (under development), outlined actions to prevent the inappropriate marketing of breast milk substitutes and unhealthy foods within their nutrition policies, while data on interventions related to such policies were captured broadly as counselling on a healthy diet, but there was no specific data on marketing of breast milk substitute and unhealthy diet. Tracking exposure to harmful information and products targeted to vulnerable groups, as well as exposure to health or nutrition information, is needed and should be incorporated into data systems.

Coverage data gaps for nutrition interventions were larger than policy and programme gaps. While coverage data availability usually depended on existing national programmes, having programmes did not ensure data availability for progress tracking. In a few instances, there was a coverage indicator for a specific intervention (e.g., iron supplementation during childhood) where no national programme was in place; coverage was extremely low in these cases, and it is possible that localised programmes or those implemented by nongovernmental entities are tracked. Nationally representative surveys in South Asia do not adequately include the full range of nutrition intervention indicators or disaggregate data sufficiently to make it possible to understand coverage and analyse issues related to inequities. The collection of systematic and comparable global data on nutrition interventions is pivotal for several reasons. First, it allows for the assessment of baseline status and changes within and across nations and regions and investigation of correlates and drivers of nutrition and nutrition transitions over time. Second, estimating the triple burden of malnutrition (i.e., undernutrition, micronutrient deficiency, and overweight and obesity) attributable to suboptimal nutrition encourages modelling, designing, and implementing specific nutrition policies and programmes to reduce disease and disparity within and across countries (Heidkamp et al., 2021). Until recently, DHS questionnaires did not adequately collect data on nutrition counselling during pregnancy, during antenatal care, and for infants (Choufani et al., 2020). Although not captured in our review, the updated DHS‐8 will allow for improved data and surveillance of nutrition intervention coverage in the next rounds of surveys.

While some indicators such as counselling coverage may be included in future rounds of population‐based surveys, other coverage indicators may not be suitable for large‐scale household surveys. For example, measurement challenges exist for specific newborn care services which mothers may have difficulty in recalling, or coverage outcomes for identification and management of SAM and MAM, which may require further refinement in measurement. Data for some interventions may need to be collected from other sources such as specialised surveys or administrative data systems. Most of the surveys in our review covered a wide range of topics including health, education, hygiene and sanitation, and few surveys focused specifically on nutrition. Resource constraints may limit the extent that nutrition interventions could be captured in all‐purpose surveys. A nutrition survey that focuses on nutrition interventions, determinants, and outcomes could help fill the data gap. However, four countries (Bangladesh, Bhutan, India, and Pakistan) have NNS, and even these surveys have data gaps. To close gaps, additional efforts are needed to examine existing data systems and allocate time and resources for data collection and analysis, and to build local capacity to interpret and use data for decision‐making/actions. Mapping of policies and programmes to coverage data is a step towards identifying and developing further strategies, as was done in the development of India's Poshan Abhiyaan nutrition data monitoring plan (Menon et al., 2020).

Our assessment of policies, programmes, and data availability was focused on indicators related to nutrition‐specific interventions, but it is equally important to assess the availability of data on nutrition‐sensitive interventions and on basic and underlying determinants of nutrition. What people eat is an important determinant of their nutritional status, but dietary intake is infrequently measured in nationally representative surveys in South Asia, particularly for women of reproductive age, and consumption of unhealthy foods is also poorly assessed (Scott et al., 2022). Though some surveys have captured counselling for consuming healthy diets, counselling on avoiding unhealthy food was only captured among adolescents in Nepal and should be expanded to other life stages. Furthermore, South Asian countries have social protection programmes that aim to improve diets such as cash or food transfers. However, measurement gaps exist for coverage of social protection schemes in South Asia (Neupane et al., 2022).

This review is not without limitations. First, our approach to reviewing policy and programming documents focused on those within the health and nutrition sector. Owing to the multisectoral and cross‐cutting nature of nutrition, it is possible that policy and programme documents in related sectors, for example, agriculture, education, and social protection, may consider nutrition in their national policies, strategies and plans, which would contribute to our findings (World Health Organization, 2013). Second, the existence of policy and programme documents does not ensure that programmes and interventions are implemented and monitored; our review did not examine implementation. Third, our review considered nutrition intervention data from primarily DHS and MICS. We recognise that there are other sources of data from administrative and nongovernmental programmatic surveys which could be leveraged to provide a more holistic picture of available nutrition data. Fourth, we recognise that our review could not adequately examine associated prevention and treatment interventions, including how countries are responding to the burden of diet‐related noncommunicable diseases, and instead relied on a proxy indicator of counselling on healthy diets.

## 5 CONCLUSIONS

Nutrition policies and programmes in South Asian countries are aligned with recommended nutrition interventions, but large gaps in available coverage data in national surveys remain. Given the increased interest in scaling up and sustaining nutrition interventions, there is a need for concerted efforts to coordinate across national and subnational levels, to ensure coherence with nutrition and quality‐related policies and standards, and to avail supporting data. Improving the availability of quality and timely nutrition data and strengthening monitoring systems is key to modelling, designing, and implementing relevant nutrition policies and programmes, and would potentially lead to programme cost savings. While our review reflected that nationally representative surveys help to provide data for monitoring nutrition intervention coverage and provide the foundation for evidence‐based recommendations to improve policy and programme actions, data gaps need to be addressed by incorporating missing indicators into the surveys or other existing data systems. Mobilising political commitment and increasing multisectoral collaboration are required for an enabling environment in support of nutrition data systems and data use for decision‐making in South Asia.

## AUTHOR CONTRIBUTIONS

Sumanta Neupane, Manita Jangid, Sunny S. Kim, Zivai Murira and Purnima Menon conceptualised the study. Sumanta Neupane and Manita Jangid conducted the literature and data reviews and analyses. Sumanta Neupane, Manita Jangid, Samuel P. Scott, and Bianca Carducci drafted and revised the manuscript; all authors contributed to the manuscript and approved the final version.

## CONFLICT OF INTEREST STATEMENT

The authors declare no conflict of interest.

## ETHICS STATEMENT

All documents and data sources used and analysed in this study are already in the public domain and freely available. Sources of the original documents and data have been acknowledged and cited.

## Supporting information

Supporting information.

## Data Availability

Data sharing is not applicable to this article as no new data were created or analysed in this study.
