# Supplementary material for: Availability of national policies, programmes, and survey‐based coverage data to track nutrition interventions in South Asia
Source: Matern Child Nutr. 2023 Aug 17;20(1):e13555. doi: 10.1111/mcn.13555 (PMC10750012; doi:10.1111/mcn.13555)
Supplement: Supplementary file 1 — Supporting information. [file MCN-20-e13555-s001.docx]

**SUPPLEMENTAL TABLE 1: Applicability of nutrition interventions across countries**

| **Life stages** | **Nutrition interventions** | **Settings** | **Applicability based on prevalence of the nutrition problem to be addressed by intervention, recommended for all settings, or existence of program in country** | | | | | | | |
| --- | --- | --- | --- | --- | --- | --- | --- | --- | --- | --- |
|  |  |  | **AF** | **BD** | **BH** | **IN** | **ML** | **NP** | **PK** | **SL** |
| Adolescence | 1. Daily or Intermittent IFA supplementation | Daily if anemia prevalence among non-pregnant women is more than 40%. Intermittent if anemia prevalence among non-pregnant women is more than 20% | 🗸  Prevalence of anemia among non-pregnant women is 40 % (Source: National Nutrition Survey (NNS) 2013) | 🗸  Prevalence of anemia among non-pregnant women is 26% (Source: Micronutrient survey: 2012) | 🗸  Prevalence of anemia among non-pregnant women is 35% (GNR country profile) | 🗸  Prevalence of anemia among non-pregnant women is 51% (Source: National Family Health Survey (NFHS) 2016) | 🗸  Prevalence of anemia among non-pregnant women is 63 % (Source: Maldives Demographic and Health Survey (MDHS) 2017) | 🗸  Prevalence of anemia among non-pregnant women is 41 % (Source: Nepal Demographic and Health Survey (NDHS) 2017) | 🗸  Prevalence of anemia among non-pregnant women is 52% % (Source: GNR country profile) | 🗸  Prevalence of anemia among non-pregnant women is 32% % (Source: GNR country profile) |
|  | 2. Preventive deworming | Where prevalence of any soil transmitted helminth infection is 20% or higher among non-pregnant women of reproductive age | 🗸  No such estimation is available but considered applicable, and country already has the program in place |  |  |  |  |  |  | 🗴  Prevalence of soil transmitted helminth is <1% (Source: National survey on intestinal nematodes-2017) |
|  | 3. Food supplementation | All countries, all settings | 🗸 | 🗸 | 🗸 | 🗸 | 🗸 | 🗸 | 🗸 | 🗸 |
|  | 4. Counseling/ education on healthy diets | All countries, all settings | 🗸 | 🗸 | 🗸 | 🗸 | 🗸 | 🗸 | 🗸 | 🗸 |
| Preconception | 5. Daily or Intermittent IFA supplementation | Daily if anemia prevalence among non-pregnant women is more than 40%. Intermittent if anemia prevalence among non-pregnant women is more than 20% | 🗸  Prevalence of anemia among non-pregnant women is 40 % (Source: NNS 2013) | 🗸  Prevalence of anemia among non-pregnant women is 26% (Source: Micronutrient survey: 2012) | 🗸  Prevalence of anemia among non-pregnant women is 35% (GNR country profile) | 🗸  Prevalence of anemia among non-pregnant women is 51% (Source: National Family Health Survey (NFHS) 2016) | 🗸  Prevalence of anemia among non-pregnant women is 63 % (Source: Maldives Demographic and Health Survey (MDHS) 2017) | 🗸  Prevalence of anemia among non-pregnant women is 41 % (Source: Nepal Demographic and Health Survey (NDHS) 2017) | 🗸  Prevalence of anemia among non-pregnant women is 52% % (Source: GNR country profile) | 🗸  Prevalence of anemia among non-pregnant women is 32% % (Source: GNR country profile) |
|  | 6. Preventive deworming | Where prevalence of any soil transmitted helminth infection is 20% or higher among non-pregnant women of reproductive age | 🗸  No such estimation is available but considered applicable, and country already has the program in place | 🗸 | 🗸 | 🗸 | 🗸 | 🗴  Prevalence of soil transmitted helminth infection among women of reproductive age is 18%. (Source: Nepal National Micronutrient Status Survey (NNMNSS) 2016) | 🗸 | 🗴  Prevalence of soil transmitted helminth is <1% (Source: National survey on intestinal nematodes-2017) |
|  | 7. Contraception | All countries, all settings | 🗸 | 🗸 | 🗸 | 🗸 | 🗸 | 🗸 | 🗸 | 🗸 |
|  | 8. Salt fortification (Iodine supplementation) | Where 20% or fewer households have access to iodized salt and pregnant women are difficult to reach | 🗸  Even though access to iodized salt is above the threshold, program exists in the country | 🗸  Even though access to iodized salt is above the threshold, program exists in the country | 🗴  91% of households have access to iodized salt. (Source: Study on IDD Elimination Sustainability in Bhutan: Establishing the country status 2010) | 🗸  Even though access to iodized salt is above the threshold, program exists in the country | 🗴  97% of households have access to iodized salt  (Source: National Micronutrient Survey 2007) | 🗸  Even though access to iodized salt is above the threshold, program exists in the country | 🗸  Even though access to iodized salt is above the threshold, program exists in the country | 🗸  Even though access to iodized salt is above the threshold, program exists in the country |
| Pregnancy | 9. Any ANC screening by a trained provider | All countries, all settings | 🗸 | 🗸 | 🗸 | 🗸 | 🗸 | 🗸 | 🗸 | 🗸 |
|  | 10. ANC screening by a trained  provider during 1st trimester | All countries, all settings | 🗸 | 🗸 | 🗸 | 🗸 | 🗸 | 🗸 | 🗸 | 🗸 |
|  | 11. Four or more ANC visits | All countries, all settings | 🗸 | 🗸 | 🗸 | 🗸 | 🗸 | 🗸 | 🗸 | 🗸 |
|  | 12. Energy and protein dietary supplementation | If underweight prevalence among women is more than 20% | 🗴  Prevalence of underweight among women is 9% (Source: NNS, 2013) | 🗸  Prevalence of underweight among women is 31% (Source: BDHS 2014) | 🗴  Prevalence of underweight among women is 11% (Source: GNR country profile) | 🗸  Prevalence of underweight among women is 23% (Source: NFHS, 2015) | 🗴  Prevalence of underweight among women is 10.7% (Source: MDHS 2017) | 🗸  Prevalence of underweight among women (15-49) is 17% (Source: NDHS 2016).  Country has food supplementation for pregnant women in some food insecure areas | 🗸  Prevalence of underweight among women is 10.7% (Source: PDHS 2018).  Country has food supplementation for pregnant women | 🗸  Prevalence of underweight among women is 9% (Source: PDHS 2018). Country has food supplementation for pregnant women |
|  | 13. Daily or intermittent IFA supplementation | All countries, all settings | 🗸 | 🗸 | 🗸 | 🗸 | 🗸 | 🗸 | 🗸 | 🗸 |
|  | 14. Vitamin A supplementation | Where 5% or more of women have a history of night-blindness in pregnancies in the past 3–5 years, or if 20% or more of pregnant women have vitamin A deficiency | 🗴  Prevalence of Vitamin A deficiency among non-pregnant women is 11% (NNS 2013) | 🗴  Data not available | 🗴  Data not available | 🗴  Data not available | 🗴  Data not available | 🗴  Prevalence of Vitamin A deficiency among non-pregnant women is 3% (NNMNSS 2016) | 🗴  Data not available | 🗴  Prevalence of Vitamin A deficiency among pregnant women is 3.4% (Source: National Nutrition and Micronutrient Survey of Pregnant Women in Sri Lanka-2015) |
|  | 15. Calcium supplementation | Where dietary calcium intake is low | 🗸 | 🗸 | 🗸 | 🗸 | 🗸 | 🗸 | 🗸 | 🗸 |
|  | 16. Iron-containing MNP supplementation | Settings with a high prevalence of nutritional deficiencies | 🗴 | 🗴 | 🗴 | 🗴 | 🗴 | 🗴 | 🗴 | 🗴 |
|  | 17. Preventive deworming | populations where pregnant women have a 20% or higher prevalence of infection with hookworm or T. trichiura infection AND a 40% or  higher prevalence of anemia | 🗸 | 🗸 | 🗸 | 🗸 | 🗸 | 🗸 | 🗸 | 🗸 |
|  | 18. Tetanus toxoid vaccination | All countries, all settings | 🗸 | 🗸 | 🗸 | 🗸 | 🗸 | 🗸 | 🗸 | 🗸 |
|  | 19. Nutritional counseling on healthy diet | All countries, all settings | 🗸 | 🗸 | 🗸 | 🗸 | 🗸 | 🗸 | 🗸 | 🗸 |
|  | 20. Weight monitoring | All countries, all settings | 🗸 | 🗸 | 🗸 | 🗸 | 🗸 | 🗸 | 🗸 | 🗸 |
|  | 21. Advice about weight after weighing | All countries, all settings | 🗸 | 🗸 | 🗸 | 🗸 | 🗸 | 🗸 | 🗸 | 🗸 |
|  | 22. Advice on consuming calcium | All countries, all settings | 🗸 | 🗸 | 🗸 | 🗸 | 🗸 | 🗸 | 🗸 | 🗸 |
|  | 23. Advice on consuming IFA | All countries, all settings | 🗸 | 🗸 | 🗸 | 🗸 | 🗸 | 🗸 | 🗸 | 🗸 |
|  | 24. Advice on consuming additional food | All countries, all settings | 🗸 | 🗸 | 🗸 | 🗸 | 🗸 | 🗸 | 🗸 | 🗸 |
|  | 25. Advice on birth preparedness | All countries, all settings | 🗸 | 🗸 | 🗸 | 🗸 | 🗸 | 🗸 | 🗸 | 🗸 |
|  | 26. Advice on exclusive breastfeeding | All countries, all settings | 🗸 | 🗸 | 🗸 | 🗸 | 🗸 | 🗸 | 🗸 | 🗸 |
| Delivery and lactation | 27. Institutional birth | All countries, all settings | 🗸 | 🗸 | 🗸 | 🗸 | 🗸 | 🗸 | 🗸 | 🗸 |
|  | 28. Skilled birth attendant | All countries, all settings | 🗸 | 🗸 | 🗸 | 🗸 | 🗸 | 🗸 | 🗸 | 🗸 |
|  | 29. Optimal timing (delayed) of umbilical cord clamping | All countries, all settings | 🗸 | 🗸 | 🗸 | 🗸 | 🗸 | 🗸 | 🗸 | 🗸 |
|  | 30. Assessment of birth weight | All countries, all settings | 🗸 | 🗸 | 🗸 | 🗸 | 🗸 | 🗸 | 🗸 | 🗸 |
|  | 31. Support for EBF and immediate skin-to-skin contact | All countries, all settings | 🗸 | 🗸 | 🗸 | 🗸 | 🗸 | 🗸 | 🗸 | 🗸 |
|  | 32. Optimal feeding of LBW infants | All countries, all settings | 🗸 | 🗸 | 🗸 | 🗸 | 🗸 | 🗸 | 🗸 | 🗸 |
|  | 33. Counseling of mothers of LBW infants on KMC | All countries, all settings | 🗸 | 🗸 | 🗸 | 🗸 | 🗸 | 🗸 | 🗸 | 🗸 |
|  | 34. PNC care for babies (3d, 7d & 6wks after delivery) | All countries, all settings | 🗸 | 🗸 | 🗸 | 🗸 | 🗸 | 🗸 | 🗸 | 🗸 |
|  | 35. PNC for women (3d, 7d & 6wks after delivery) | All countries, all settings | 🗸 | 🗸 | 🗸 | 🗸 | 🗸 | 🗸 | 🗸 | 🗸 |
|  | 36. Breastfeeding counseling | All countries, all settings | 🗸 | 🗸 | 🗸 | 🗸 | 🗸 | 🗸 | 🗸 | 🗸 |
|  | 37. IFA supplementation | with a 20% or higher population prevalence of gestational anemia | 🗸  38% of pregnant women are anemic (Source: GNR country profile) | 🗸  50% of pregnant women are anemic (BDHS 2011) | 🗸  27% of pregnant women are anemic (Source: NNS 2015) | 🗸  50% of pregnant women are anemic (Source: GNR country profile) | 🗸  47% of pregnant women are anemic (Source: GNR country profile) | 🗸  27% of pregnant women are anemic (Source: NNMSS 2016) | 🗸  51% of pregnant women are anemic (Source: GNR country profile) | 🗸  32% of pregnant women are anemic (Source: MNS 2015) |
|  | 38. Food supplementation for malnourished lactating women | All countries, all settings | 🗸 | 🗸 | 🗸 | 🗸 | 🗸 | 🗸 | 🗸 | 🗸 |
| Childhood | 39. Breastfeeding counseling | All countries, all settings | 🗸 | 🗸 | 🗸 | 🗸 | 🗸 | 🗸 | 🗸 | 🗸 |
|  | 40. Counseling on appropriate complementary feeding | All countries, all settings | 🗸 | 🗸 | 🗸 | 🗸 | 🗸 | 🗸 | 🗸 | 🗸 |
|  | 41. Food supplementation for complementary feeding | In food insecure populations | 🗸 | 🗸 | 🗴  No food insecurity | 🗸 | 🗸 | 🗸 | 🗸 | 🗸 |
|  | 42. Iron-containing MNP supplementation | in which the prevalence of anemia in children under 5 years of age is 20% or more | 🗸  Prevalence of anemia among children 6-59 months is 45% (Source: NNS 2013) | 🗸  Prevalence of anemia among children 6-59 months is 33% (Source: MNS 2012) | 🗸  Prevalence of anemia among children 6-59 months is 43% (Source: NNS 2015) | 🗸  Prevalence of anemia among children 6-59 months is 58% (Source: NFHS 2015) | 🗸  Prevalence of anemia among children 6-59 months is 50% (Source: MDHS 2016) | 🗸  Prevalence of anemia among children 6-59 months is 53% (Source: NDHS 2016) | 🗸  Prevalence of anemia among children 6-59 months is 58% (Source: NNS 2018) | 🗸  Prevalence of anemia among children 6-59 months is 15% (Source: MNS 2015).  Country has program |
|  | 43. Daily IFA supplementation | Daily if anemia prevalence among children aged 6-59 months is 40% or more.  Intermittent for children agen 24-59 months if anemia prevalence among this gourp is 20% or more. | 🗸  Prevalence of anemia among children 6-59 months is 45% (Source: NNS 2013) | 🗸  Prevalence of anemia among children 6-59 months is 33% (Source: MNS 2012) | 🗸  Prevalence of anemia among children 6-59 months is 43% (Source: NNS 2015) | 🗸  Prevalence of anemia among children 6-59 months is 58% (Source: NFHS 2015) | 🗸  Prevalence of anemia among children 6-59 months is 50% (Source: MDHS 2016) | 🗸  Prevalence of anemia among children 6-59 months is 53% (Source: NDHS 2016) | 🗸  Prevalence of anemia among children 6-59 months is 58% (Source: NNS 2018) | 🗴  Prevalence of anemia among children 6-59 months is 15% (Source: MNS 2015) |
|  | 44. Zinc supplementation during diarrhea | All countries, all settings | 🗸 | 🗸 | 🗸 | 🗸 | 🗸 | 🗸 | 🗸 | 🗸 |
|  | 45. ORS during diarrhea | All countries, all settings | 🗸 | 🗸 | 🗸 | 🗸 | 🗸 | 🗸 | 🗸 | 🗸 |
|  | 46. Vitamin A supplementation | Where the prevalence of night blindness is 1% or more in children aged 24–59 months, or the prevalence of vitamin A deficiency is 20% or higher in infants and children aged 6–59 months | 🗸  Vitamin A deficiency is 50% among children 6-59 months (Source: NNS, 2013) | 🗸  Vitamin A deficiency is 20% among children 6-59 months (Source: MNS, 2012) | 🗸  No data available, but country has program | 🗸  Vitamin A deficiency is 20% among children 6-59 months (Source: CNNS, 2018) | 🗸  No data available, but considered applicable as government has the program | 🗸  Vitamin A deficiency is 4.2% among children 6-59 months (Source: NNMSS, 2016) | 🗸  Vitamin A deficiency is 51% among children 6-59 months (Source: NNS, 2018) | 🗸  Vitamin A deficiency is 29% among children 6-59 months (Source: MNS, 2012) |
|  | 47. Preventive deworming | Living in areas where the baseline prevalence of any soil-transmitted infection is 20% or higher among children aged 12 months  and older | 🗸 | 🗸 | 🗸 | 🗸 | 🗸 | 🗸 | 🗸 | 🗸 |
|  | 48. Growth monitoring (weight assessment) | All countries, all settings | 🗸 | 🗸 | 🗸 | 🗸 | 🗸 | 🗸 | 🗸 | 🗸 |
|  | 49. Counseling on nutritional status | All countries, all settings | 🗸 | 🗸 | 🗸 | 🗸 | 🗸 | 🗸 | 🗸 | 🗸 |
|  | 50. Identification of severe or moderate underweight | All countries, all settings | 🗸 | 🗸 | 🗸 | 🗸 | 🗸 | 🗸 | 🗸 | 🗸 |
|  | 51. Inpatient management of SAM | All countries, all settings | 🗸 | 🗸 | 🗸 | 🗸 | 🗸 | 🗸 | 🗸 | 🗸 |
|  | 52. Outpatient management of SAM | All countries, all settings | 🗸 | 🗸 | 🗸 | 🗸 | 🗸 | 🗸 | 🗸 | 🗸 |
|  | 53. Management of MAM | All countries, all settings | 🗸 | 🗸 | 🗸 | 🗸 | 🗸 | 🗸 | 🗸 | 🗸 |
|  | 54. Immunization | All countries, all settings | 🗸 | 🗸 | 🗸 | 🗸 | 🗸 | 🗸 | 🗸 | 🗸 |
| 🗸=Applicable; 🗴=Not applicable | | | | | | | | | | |

**SUPPLEMENTAL TABLE 2: Nutrition-relevant policies and program documents reviewed to assess policy and program availability by country**

| **Country** | **Documents** |
| --- | --- |
| Afghanistan | 1. National Maternal, Infant and Young Child Nutrition Policy 2019–2023 (Afghanistan, Ministry of Public Health 2019) 2. School Health Policy 2019–2029 (Afghanistan, Ministry of Education 2019) 3. National Reproductive, Maternal, Newborn, Child and Adolescent Health Strategy (RMNCAH) 2017–2021 (Afghanistan, Ministry of Public Health 2017) 4. National Public Nutrition Strategy (NPNS) 2019–2023 (Afghanistan, Ministry of Public Health 2019) 5. National Maternal Infant and Young Child Nutrition Strategy (MIYCN) 2019–2023 (Afghanistan, Ministry of Public Health 2019) 6. Operational Guide for Implementing the National Maternal, Infant and Young Child Nutrition Strategy 2019–2023 (Afghanistan, Ministry of Public Health 2019) 7. Integrated Management of Acute Malnutrition: National Guidelines 2018 (Afghanistan, Ministry of Public Health 2018) 8. A Basic Package of Health Services for Afghanistan 2010 (Afghanistan, Ministry of Public Health 2010) 9. Mother and Child Health Handbook (Afghanistan, Ministry of Public Health 2019) 10. The Essential Package of Hospital Services for Afghanistan (Afghanistan, Ministry of Public Health 2005) |
| Bangladesh | 1. Operational Plan for National Nutrition Services (NNS) 2017–2022 (Bangladesh, Directorate General of Health Services 2017b) 2. National Strategy on Prevention and Control of Micronutrient Deficiencies, Bangladesh (2015–2024 (Bangladesh, Directorate General of Health Services 2015) 3. Second National Plan of Action for Nutrition: 2016–2025 (Bangladesh, Ministry of Health and Family Welfare 2017) 4. National Immunization Policy (Bangladesh, Directorate General of Health Services 2014) 5. Bangladesh Essential Health Service Package (ESP) (Bangladesh, Ministry of Health and Welfare 2016a) 6. 4th Health, Population and Nutrition Sector Programme: Operational Plan, (OP) (January 2017–June 2020): Maternal Neonatal Child and Adolescent Health (Bangladesh, Directorate General of Health Services 2017a) 7. National Nutrition Policy 2015 (Bangladesh, Ministry of Health and Family Welfare 2015) 8. National Strategy for Adolescent Health 2017–2030 (Bangladesh, Ministry of Health and Welfare 2016c) 9. National Strategy for Infant and Young Child Feeding in Bangladesh (Bangladesh, Institute of Public Health Nutrition 2007) 10. Bangladesh National Strategy for Maternal Health 2019–2030 (Bangladesh, Ministry of Health and Family Welfare 2019) 11. National Neonatal Health Strategy and Guidelines for Bangladesh (Bangladesh, Ministry of Health and Welfare 2009) 12. Clinical Guidelines on Infant and Young Child Feeding (IYCF) (Alive & Thrive, n.d.) 13. Operational Guideline for Adolescents Nutrition Interventions 2020 (Bangladesh, Institute of Public Health Nutrition 2020) 14. National Guidelines for the Facility-Based Management of Children with Severe Acute Malnutrition in Bangladesh (Bangladesh, Institute of Public Health Nutrition 2017) 15. National Guidelines for the Management of Severely Malnourished Children in Bangladesh (Bangladesh, Institute of Public Health Nutrition 2008) 16. National Guidelines for Community Based Management of Acute Malnutrition in Bangladesh (Bangladesh, Institute of Public Health Nutrition 2011) |
| Bhutan | 1. Infant and Young Child Feeding Practice Policy of Bhutan, 2015—Draft (Bhutan, Ministry of Health 2015) 2. Strategy for Control of Iron Deficiency in Bhutan (Final Draft) (Bhutan, Ministry of Health 2018) 3. National Reproductive Health Strategy of Bhutan (2018–2023) (Bhutan, Department of Public Health 2018) 4. Bhutan Every Newborn Action Plan (2016–2023) (Bhutan, Department of Public Health, UNICEF, and WHO 2016) 5. Food and Nutrition Security Policy of the Kingdom of Bhutan, 2014 (Bhutan, Royal Government of Bhutan 2014) 6. National Food and Nutrition Security Strategy (2016–2025) and Action Plan (2016–2018) (Bhutan, Royal Government of Bhutan 2016) 7. Mother & Child Health Handbook (Bhutan, Ministry of Health 2019) 8. EPI Services Manual for Health Workers (Bhutan, Department of Public Health 2014) 9. Accelerating Actions for Reducing Under Nutrition and Micronutrient Deficiencies Among Women and Children (Bhutan, National Nutrition Task Force, n.d.) |
| India | 1. Intensified National Iron plus Initiative (I-NIPI) (India, Ministry of Health and Family Welfare 2018) 2. National Nutrition Strategy (India, NITI Aayog 2017) 3. Directives under section 16 (5) of Food Safety and Standards Act, 2006 regarding operationalisation of the Food Safety and Standards (Food Products Standards and Food Additive) Amendment Regulations (India, Ministry of Health and Family Welfare 2020) 4. Guidelines for Antenatal Care and Skilled Attendance at Birth by ANMs/LHVs/SNs (India, Ministry of Health and Family Welfare 2010) 5. Operational Guidelines for Food Safety and Hygiene for Supplementary Nutrition under ICDS (India, Ministry of Women and Child Development 2013) 6. National Guidelines for Calcium Supplementation During Pregnancy and Lactation (India, Maternal Health Division, Ministry of Health and Family Welfare 2014) 7. National Guidelines on Infant and Young Child Feeding (India, Ministry of Human Resource Development 2004) 8. Guidelines for Janani-Shishu Suraksha Karyakram (JSSK) (India, Maternal Health Division, Ministry of Health and Family Welfare 2011) 9. Janani Suraksha Yojana (India, Ministry of Health and Family Welfare 201 10. India Newborn Action Plan (INAP) (India, Ministry of Health and Family Welfare 2014) 11. Guidelines for enhancing optimal infant and young child feeding practices, 2013 12. Operational Guidelines for Intensified Diarrhoea Control Fortnight (India, Ministry of Health and Family Welfare 2017) 13. Guidelines on mandatory use of Double Fortified Salt (DFS) in National Programmes ICDS (India, Ministry of Women and Child Development 2011) 14. Guidelines on Vitamin A Supplementation Program (India, Ministry of Health and Family Welfare 2006) 15. Directives on growth monitoring (India, Ministry of Women and Child Development & Ministry of Women and Child Development 2010) 16. Operational Guidelines for Facility Based Management of Children with Severe Acute Malnutrition (India, Ministry of Health and Family Welfare 2011) 17. Operational Guidelines for Intensified Mission Indradhanush (India, Ministry of Health and Family Welfare 2017) |
| Maldives | 1. Multi-Sectoral Action Plan for the Prevention and Control of Noncommunicable Diseases in Maldives (2014-2020) (Ministry of Health and WHO Country Office, 2014) 2. Integrated National Nutrition Strategic Plan 2013–2017 (Maldives, Ministry of Health 2016a) 3. Health Master Plan (Maldives, Ministry of Health 2014) 4. National Reproductive Health Strategy 2014–2018 (Maldives, Ministry of Health, n.d.) 5. National Child Health Strategy—Every Newborn Action Plan 2016–2020 (Maldives, Ministry of Health 2016b) 6. Social and Behavior Change Communication Strategy: The First 1000 Days Matter (2019–2021) (UNICEF and Maldives, Ministry of Health 2019) 7. Immunization Handbook for Health Care Professionals (National Program on Immunization 2015) |
| Nepal | 1. National Nutrition Policy and Strategy (NNPS) (Nepal, Child Health Division, Department of Health Services, Ministry of Health and Population 2004) 2. National Strategy for the Control of Anemia Among Women and Children in Nepal (Nepal, Department of Health Services 2002) 3. National Policy on Skilled Birth Attendants (Nepal, Ministry of Health and Population 2006) 4. Strategy for Infant and Young Child Feeding: Nepal 2014 (Nepal, Ministry of Health and Population 2014) 5. Multi-Sector Nutrition Plan 2018–2022 (Nepal, National Planning Commission 2017) 6. National Safe Motherhood Plan 2002–2017 (Nepal, Family Health Division 2002) 7. Nepal's Every Newborn Action Plan (Nepal, Ministry of Health 2016) 8. Multisectoral Action Plan for the Prevention and Control of Non Communicable Diseases (2014-2020) (Government of Nepal and WHO Country Office Nepal 2014 9. Growth Monitoring Guideline (Nepal, Child Health Division, n.d.) 10. Guidelines for Weekly Iron Folic Acid Supplementation for Adolescent girls (Nepal, Department of Child Health 2016) 11. Comprehensive Nutrition-Specific Intervention Training Package for Health Workers (CNSI) (Nepal, Ministry of Health 2019) 12. NEPAL Integrated Management of Acute Malnutrition (IMAM) Guideline (UNICEF and Nepal, Child Health Division 2017) 13. Essential Package of Health Services Country Snapshot: Nepal (Wright 2015) |
| Pakistan | 1. National Action Plan for Prevention and Control of Non-Communicable Diseases and Health Promotion in Pakistan (Ministry of Health, WHO Pakistan Office and Heartlife 2004) 2. National IRMNCAH&N Strategy (2016–2020) (Pakistan, Ministry of Health 2017) 3. Pakistan Multi-Sectoral Nutrition Strategy (2018–2025) (Ministry of Planning, Development and Reform 2018) 4. Pakistan Infant and Young Child Feeding Strategy, 2016–2020 (Pakistan, Ministry of National Health Services, Regulation and Coordination 2015) 5. National Vision 2016–2025 for Coordinated Priority Actions (Pakistan, Ministry of National Health Services, Regulation and Coordination 2016) 6. Pakistan Adolescent Nutrition Strategy (Pakistan, Ministry of Health Services, Regulations and Coordination, gain, WHO and UNICEF 2020) 7. National Guidelines for the Management of Acute Malnutrition Among Children Under Five and Pregnant and Lactating Women (Pakistan, Ministry of Health 2009) revised 2015 8. National Maternal, Neonatal and Child Health Program (Pakistan, Ministry of Health 2007) 9. Essential Package of Health Services Country Snapshot: Pakistan (Wright 2015) 10. Adolescent Nutrition and Supplementation Guidelines for Pakistan (Pakistan, Ministry of National Health Services, Regulations and Coordination and WHO 2019) |
| Sri Lanka | 1. National Nutrition Policy of Sri Lanka (Sri Lanka, Ministry of Healthcare and Nutrition 2010a) 2. The National Policy and Strategic Framework for Prevention and Control of Chronic Non-Communicable Diseases (Sri Lanka Ministry of Health 2010b) 3. National Policy on Maternal and Child Health (Sri Lanka, Ministry of Health 2012a) 4. Multi Sector Action Plan for Nutrition 2018–2025 (Sri Lanka, National Nutrition Secretariat, n.d.) 5. National Strategy for Infant and Young Child Feeding, Sri Lanka (2015–2020) (Sri Lanka, Ministry of Health, Nutrition and Indigenous Medicine, n.d.) 6. National Strategic Plan on Adolescent and Youth Health (2018–2025) (Sri Lanka, Family Health Bureau 2018) 7. Strategies to Promote Optimal Fetal Growth and to Minimze the Prevalance of Low Birth Weight in Sri Lanka: Health Sector Response (Sri Lanka, Family Health Bureau 2013) 8. National Strategic Plan on Child Health in Sri Lanka (2018-2025) (Sri Lanka, Family Health Bureau 2016a) 9. National Multisectoral Action Plan for the Prevention and Control of Noncommunicable Diseases (2016-2020) (Sri Lanka, Ministry of Health 2016b) 10. National Strategic Plan Maternal and Newborn Health (2017-2025) (Sri Lanka, Family Health Bureau 2017) 11. Vitamin A Megadose Supplementation – Revised Schedule (General Circular No. 01–02/2009) (Sri Lanka, Ministry of Health Care and Nutrition 2009) 12. Maternal Care Package: A Guide to Field Healthcare Workers (Sri Lanka, Family Health Bureau 2011) 13. Management of Severe and Moderate Acute Undernutrition of the Children Under Five Years of Age: Manual for Health Workers in Sri Lanka (Sri Lanka, Ministry of Health, Nutrition and Indigenous Medicine 2019) 14. National Guidelines for Newborn Care (Volume 1) (Sri Lanka, Ministry of Health 2014) 15. Zinc (Zn) Supplementation in Managing Diarrhoea Among Children Under Five Years of Age (General Circular No. 02–161/2013) (Sri Lanka, Ministry of Health 2013) 16. Guidelines on Infant and Young Child Feeding (Sri Lanka, Ministry of Health 2007) 17. Guidelines on De-worming Children and Pregnant Women Against Soil Transmitted Helminths in Community Setting 2019–2022 (General Circular No. 01–58/2018) (Sri Lanka, Ministry of Health, Nutrition and Indigenous Medicine 2018) 18. Micronutrient Supplementation for School Children From the Year 2019 Onwards (General Circular No. 01–12/2019) (Sri Lanka, Ministry of Health, Nutrition and Indigenous Medicine 2019) 19. School Health Programme (Sri Lanka, Office of the Secretary Health, Nutrition and Indigenous Medicine 2016) 20. Iron supplementation for infants and young children (General Circular No. 01–68/2016) (Sri Lanka, Ministry of Health 2016) 21. Strengthening Postpartum Family Planning Services Provided by Curative Institutions (General Circular No: 01–45/2017) (Sri Lanka, Ministry of Health, Nutrition and Indigenous Medicine) 22. Protocol for managing nutrition problems among under five children in the community (General Circular No. 02–18/2008) (Sri Lanka, Ministry of Health 2008) |

**SUPPLEMENTAL TABLE 3: Nationally representative population-based surveys reviewed to assess coverage data availability by country**

| **Countries** | **Surveys** |
| --- | --- |
| Afghanistan | Afghanistan Demographic and Health Survey (ADHS) 2015  National Nutrition Survey (NNS) 2013 |
| Bangladesh | Multiple Indicator Cluster Survey (MICS) 2019  Bangladesh Demographic and Health Survey (BDHS) 2017-18  Bangladesh Demographic and Health Survey (BDHS) 2014 |
| Bhutan | National Nutrition Survey (NNS) 2015  Multiple Indicator Cluster Survey (MICS) 2010 |
| India | Comprehensive National Nutrition Survey (CNNS) 2019  National Family Health Survey (NFHS) 2016 |
| Maldives | Maldives Demographic and Health Survey (MDHS) 2017  National Micronutrient Survey (NMNS) 2010 |
| Nepal | Nepal Demographic and Health Survey (NDHS) 2016  Nepal National Micronutrient Status Survey (2016) |
| Pakistan | Pakistan Demographic and Health Survey (PDHS) 2017  National Nutrition Survey (NNS) 2018 |
| Sri Lanka | Sri Lanka Demographic and Health Survey (PDHS) 2016  National Nutrition and Micronutrient Survey (NNMS) 2012 (Sri Lanka Ministry of Health and UNICEF, 2012)  National Nutrition Survey of Lactating Women in Sri Lanka (NNSLW) 2017 (Medical Research institute, UNICEF, and WFP, 2017a)  National Nutrition and Micronutrient Survey of Pregnant Women in Sri Lanka (NNMSPW) 2015 (Medical Research institute, UNICEF, and WFP, 2017b)  NNMS among school adolescents aged ten to18 years in Sri Lanka 2019 (Medical Research Institute, 2019) |
